# Functional Characterization of a First Avian Cytochrome P450 of the CYP2D Subfamily (CYP2D49)

**DOI:** 10.1371/journal.pone.0038395

**Published:** 2012-06-04

**Authors:** Hua Cai, Jun Jiang, Qi Yang, Qingmei Chen, Yiqun Deng

**Affiliations:** College of Life Sciences, South China Agricultural University, Guangzhou, Guangdong, People's Republic of China; University of South Florida College of Medicine, United States of America

## Abstract

The CYP2D family members are instrumental in the metabolism of 20–25% of commonly prescribed drugs. Although many CYP2D isoforms have been well characterized in other animal models, research concerning the chicken CYP2Ds is limited. In this study, a cDNA encoding a novel *CYP2D* enzyme (*CYP2D49*) was cloned from the chicken liver for the first time. The *CYP2D49* cDNA contained an open reading frame of 502 amino acids that shared 52%–57% identities with other CYP2Ds. The gene structure and neighboring genes of *CYP2D49* are conserved and similar to those of human *CYP2D6*. Additionally, similar to human CYP2D6, CYP2D49 is un-inducible in the liver and expressed predominantly in the liver, kidney and small intestine, with detectable levels in several other tissues. Metabolic assays of the CYP2D49 protein heterologously expressed in *E. coli* and Hela cells indicated that CYP2D49 metabolized the human CYP2D6 substrate, bufuralol, but not debrisoquine. Moreover, quinidine, a potent inhibitor of human CYP2D6, only inhibited the bufuralol 1′-hydroxylation activity of CYP2D49 to a negligible degree. All these results indicated that CYP2D49 had functional characteristics similar to those of human CYP2D6 but measurably differed in the debrisoquine 4′-hydroxylation and quinidine inhibitory profile. Further structure-function investigations that employed site-directed mutagenesis and circular dichroism spectroscopy identified the importance of Val-126, Glu-222, Asp-306, Phe-486 and Phe-488 in keeping the enzymatic activity of CYP2D49 toward bufuralol as well as the importance of Asp-306, Phe-486 and Phe-488 in maintaining the conformation of CYP2D49 protein. The current study is only the first step in characterizing the metabolic mechanism of CYP2D49; further studies are still required.

## Introduction

Cytochrome P450 monooxygenases (CYPs) are heme-containing enzymes that are responsible for metabolizing numerous endogenous and exogenous compounds, including the steroid hormones, drugs, carcinogens and environmental pollutants [1]. In humans, the CYP2D enzymes are particularly important in drug metabolism. Members of the CYP2D family constitute 2–4% of the total hepatic CYPs but are responsible for the metabolism of 20–25% of commonly prescribed drugs [2]. Furthermore, CYP2Ds exhibit extensive genetic polymorphism, many types are associated with altered or abolished enzyme activities, which leads not only to severe adverse effects in clinical therapy but also to a non-response to medications [3]–[5].

A large amount of experimental evidences have indicated the functional importance of CYP2D6 and its variants in humans [6], [7]. Moreover, the crystal structure of human CYP2D6 has been solved and many important amino acid residues implicated in substrate recognition and binding have been determined, including Phe-120, Glu-216, Asp-301, Phe-481 and Phe-483 [8], [9]. Additionally, many isoforms of the CYP2D subfamily other than human CYP2D6 have been well characterized in animal models of drug development, such as rats [10]–[13], mice [14], [15], guinea pigs [16], dogs [17], [18], bovines [19], rabbits [20] and monkeys [21], [22]. However, most investigations have focused on their sequence clonings, biochemical characterizations and catalytic activities; little is known about their catalytic mechanisms or the relationships between their functions and structures.

The chicken is one of the most common domestic animals [23]. Defining the contribution of a chicken CYP isoform to the metabolism of a specific drug is important not only for poultry pharmacology and toxicology but also for human health because of the possible presence of poisonous drug metabolites in chicken products, such as meat and eggs. To date, several important CYPs, such as *CYP1A4/5* [24], *CYP3A37* [25] and *CYP2C45* [26], have been cloned and identified in chickens. However, little is known about the CYP2D isoforms; though studies with chicken liver microsomes have suggested that CYP2D isoforms are present in chickens based on the observed metabolism of bufuralol, which is a prototypical substrate of human CYP2D6 [27].

Here, we describe the cloning, characterization and catalytic functional studies of a novel chicken *CYP2D* gene, termed *CYP2D49*, which was the first *CYP2D* gene reported in chickens. Through studies of its sequence, tissue expression pattern and inductive properties as well as metabolic characterization, we found that CYP2D49 had the ability to catalyze bufuralol 1′-hydroxylation and may be a counterpart to human CYP2D6 in chickens. Furthermore, we studied the catalytic mechanism of CYP2D49 toward bufuralol and elucidated the key amino acid residues involved in its enzymatic activity and protein conformation.

## Results

### Identification of a novel *CYP2D* gene from the chicken liver with similarity to human *CYP2D6*

**Figure 1:**
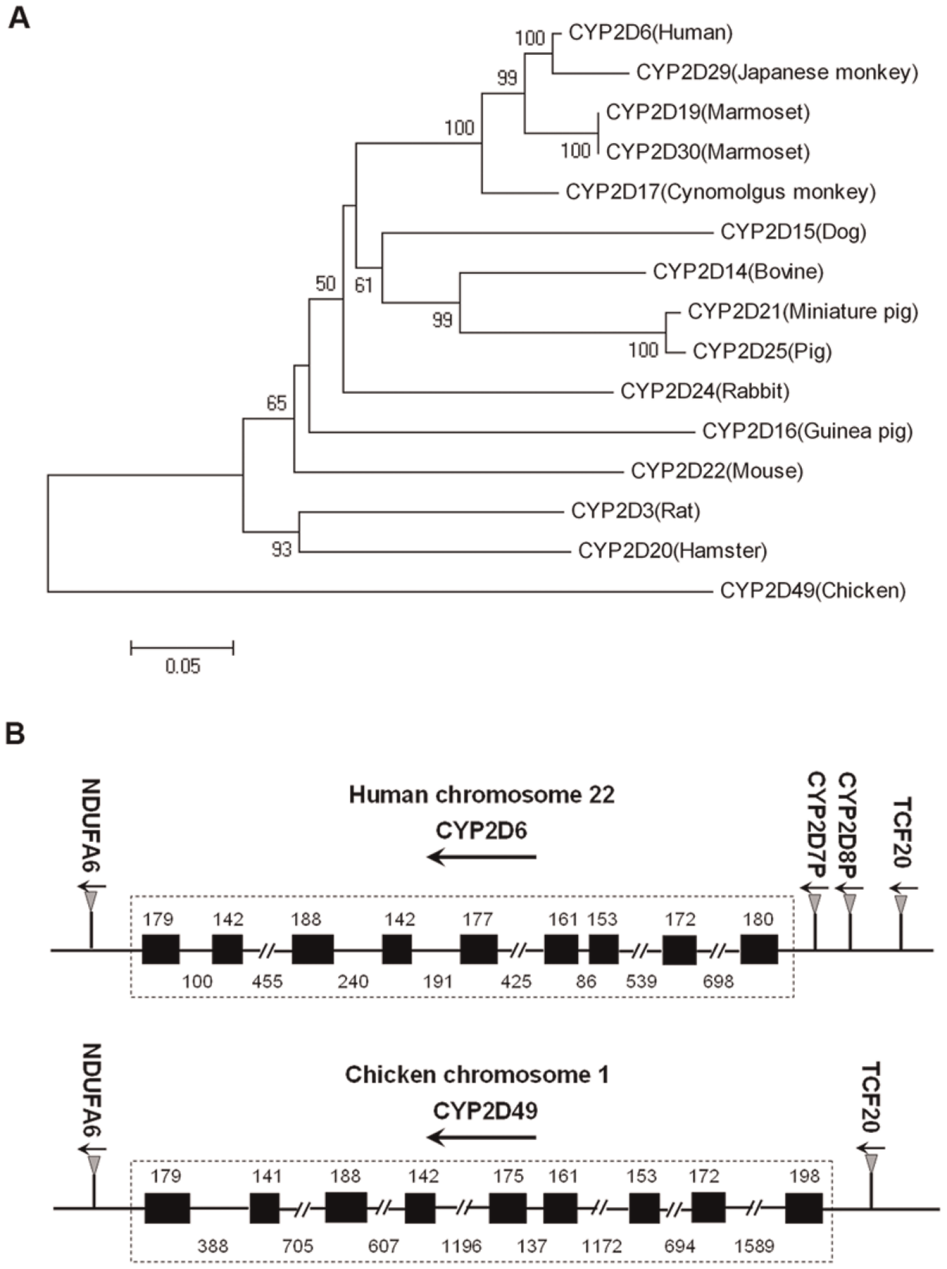
Phylogenetic tree of CYP2D amino acid sequences and genomic structures of human *CYP2D6* and chicken *CYP2D49*. (A) Phylogeny of CYP2D amino acid sequences from the chicken and other animal species. The neighbor-joining tree was created using the Molecular Evolutionary Genetics Analysis Version 4 software. The numbers on the branches indicate the number of times per 100 bootstrap replicates that the branch appeared in the trees, estimated by a random resampling of the data. Only bootstrap values greater than 50% are shown. The scale bar represents 5 substitutions in 100 residues. (B) Genomic structures of human *CYP2D6* and chicken *CYP2D49*. The diagram of the organization of the *CYP2D* subfamily in humans and chickens was determined by the BLAT analysis of the human and chicken genome data from NCBI database. Exons are indicated by boxes, whereas introns are indicated by lines. The lengths of the exons and introns are expressed in base pairs. The arrowheads indicate the direction of transcription.

To identify the CYP2D isoforms in chickens, we first searched against the chicken EST database with the human *CYP2D6* sequence. This resulted in the discovery of a unique unpublished cDNA clone (GenBank accession no. CR354312.1). Based on this discovery, we cloned the coding sequence of the cDNA (GenBank accession no. JQ241277) from the chicken liver for the first time. The sequence has been submitted to the Committee on P450 Nomenclature, which suggested the name *CYP2D49* for this gene [25], [28]. To demonstrate the genetic distance between chicken CYP2D49 and other CYP2D isoforms in experimental animal species, the identities of the respective CYP2D isoforms are indicated as percentages in Table 1. The deduced 502 amino acid residues sequence of CYP2D49 possessed 52%–57% identities with the other CYP2D enzymes (Table 1). This finding, together with a phylogenetic comparison of the deduced amino acid sequence of CYP2D49 with the sequences of other CYP2D isoforms (Fig. 1A), indicated that chicken CYP2D49 belonged to the CYP2D family, but showed far evolutionary distance to other CYP2D enzymes because of the remarkable interspecies differences.

**Table 1:** Comparison of the deduced amino acid sequence of CYP2D49 with the sequences of other CYP2D isoforms.

Species	Forms	Identity	Accession No.
Chicken	CYP2D49	100%	AEZ51809
Rat	CYP2D3	57%	J02868
Human	CYP2D6	56%	M33388
Bovine	CYP2D14	57%	X68481
Dog	CYP2D15	54%	D17397
Guinea pig	CYP2D16	52%	U21486
Cynomolgus monkey	CYP2D17	56%	U38218
Marmoset	CYP2D19	55%	D29822
Hamster	CYP2D20	56%	D86476
Miniature pig	CYP2D21	57%	D89502
Mouse	CYP2D22	53%	AF221525
Rabbit	CYP2D24	53%	AB008785
Pig	CYP2D25	56%	Y16417
Japanese monkey	CYP2D29	55%	AF301911
Marmoset	CYP2D30	55%	AY082602

To determine the genomic structure of chicken *CYP2D49*, we further searched the chicken genomic database with the *CYP2D49* sequence and compared the gene structure and the neighboring genes of chicken *CYP2D49* to those of human *CYP2D6*. As shown in Fig. 1B, *CYP2D49* localized in chromosome 1 and is composed of nine exons and eight introns; additionally, its exon–intron organization as well as the corresponding sizes of these segments and the coding region boundaries are conserved and similar to those of human *CYP2D6* (Fig. 1B). Furthermore, we found a set of genes neighboring *CYP2D49* that demonstrate highly conserved synteny to human *CYP2D6* (Fig. 1B). These results suggest that *CYP2D49* may be the chicken counterpart to human *CYP2D6*.

### Expression of recombinant CYP2D49 protein and detection of the specificity of anti-CYP2D49 antiserum

**Figure 2:**
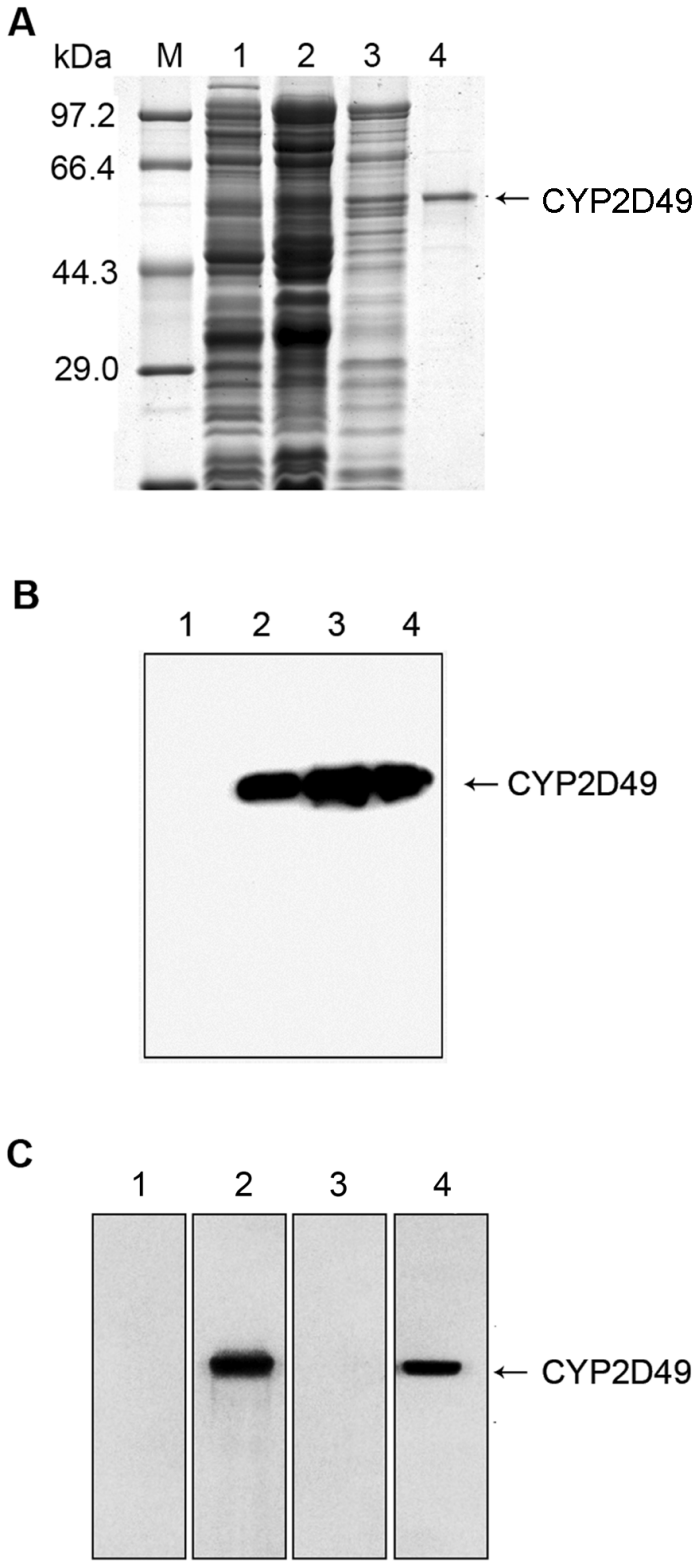
Expression of recombinant CYP2D49 protein and detection of the specificity of anti-CYP2D49 antiserum. (A) Proteins were analyzed by SDS-PAGE on 10% Tris-Glycine gels stained with Coomassie brilliant blue R-250. Lane 1: non-IPTG-induced bacteria; lane 2: IPTG-induced total cell lysates; lane 3: supernatant of the IPTG-induced cell lysates and lane 4: the protein purified by Ni^2+^-NTA affinity chromatography. (B) Western blotting analysis was used to confirm whether the prokaryotic expression of CYP2D49 protein had succeeded. The protein samples described in Fig. 2A were detected by the anti-Myc antibody. Lane 1: non-IPTG-induced bacteria; lane 2: IPTG-induced total cell lysates; lane 3: supernatant of the IPTG-induced cell lysates and lane 4: the protein purified by Ni^2+^-NTA affinity chromatography. (C) The protein samples extracted from Hela cells which were transiently transfected with pcDNA-*CYP2D49* were separated by SDS-PAGE and immunoblotted using different antibodies. Lane 1: the normal mouse serum; lane 2: the polyclonal anti-CYP2D49 antiserum; lane 3: the anti-CYP2D49 antiserum pre-adsorbed with excess antigen (purified CYP2D49 protein); lane 4: the anti-Myc antibody.

To study the function of the CYP2D49 protein, the recombinant protein was expressed in a prokaryotic expression system. As shown in Fig. 2A, a Myc-His-tagged fusion CYP2D49 protein of approximately 55 kDa was expressed (Lanes 2 and 3) and successfully purified by Ni^2+^-NTA affinity chromatography (Lane 4). Western blotting detection using an anti-Myc antibody further confirmed the correction of the recombinant expression (Fig. 2B). To further characterize the expression patterns of CYP2D49 at the protein level, a polyclonal anti-CYP2D49 antiserum was generated by immunizing mice with the purified CYP2D49 protein. Western blotting results showed that this anti-CYP2D49 antiserum (Fig. 2C, lane 2), but not the pre-immune mouse serum (Fig. 2C, lane 1), was able to recognize over-expressed CYP2D49 to provide a specific band at 55 kDa. Moreover, the band is recognized by the anti-Myc antibody (Fig. 2C, lane 4) but not by the anti-CYP2D49 antiserum that had been pre-adsorbed with excess antigen (purified CYP2D49-Myc-His-tag fusion protein) (Fig. 2C, lane 3). These results indicate that the generated antiserum can recognize CYP2D49 with high specificity.

### *In vivo* and *in vitro* expression patterns of CYP2D49

**Figure 3:**
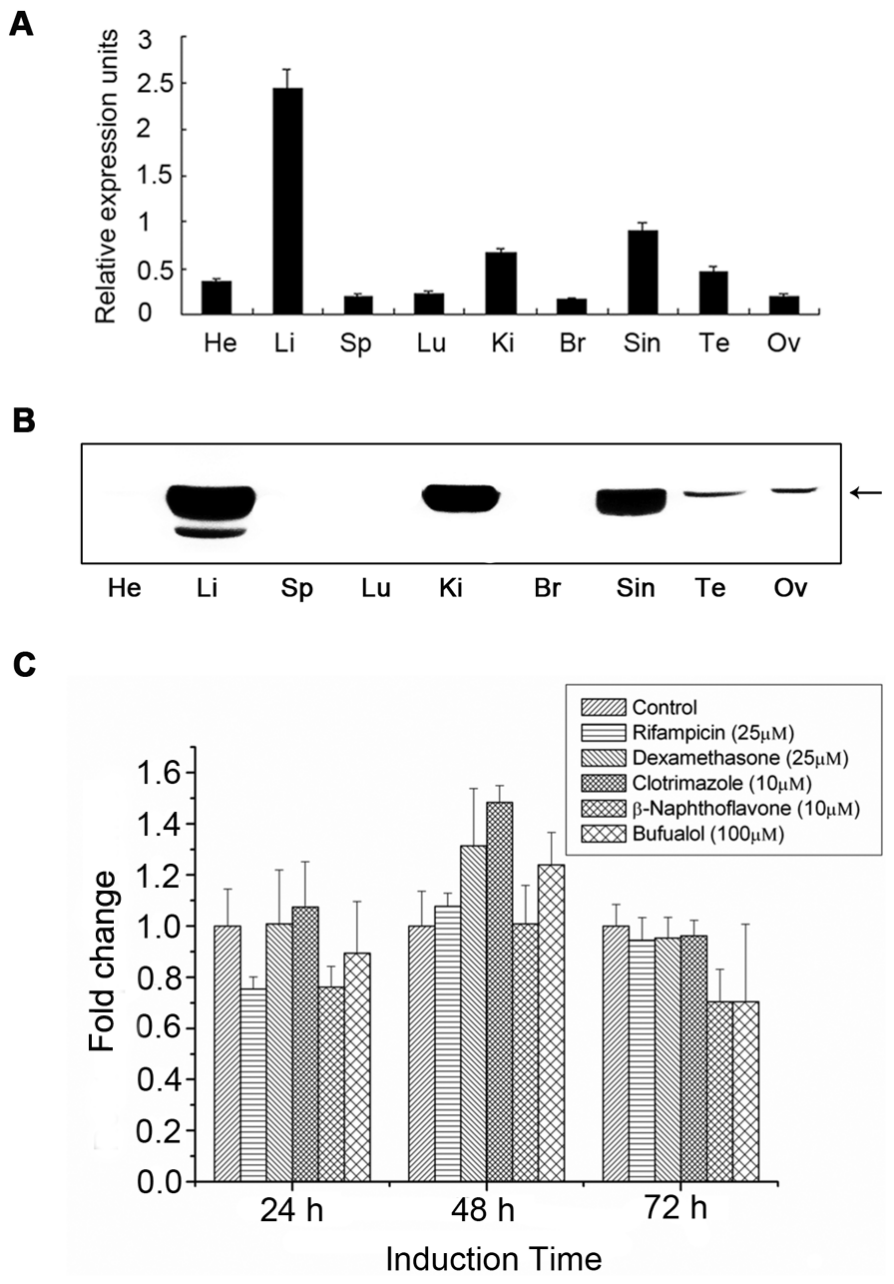
*In vivo* and *in vitro* expression patterns of CYP2D49. (A) *CYP2D49* transcripts in the indicated tissues of the healthy chicken were detected by real-time PCR amplification. The expression of the target gene was calculated relative to that of *18S rRNA* according to the 2^−ΔCT^ method. Error bars represent standard deviations obtained by measuring each sample in triplicate. He-Heart; Li-Liver; Sp-Spleen; Lu-Lung; Ki-Kidney; Br-Brain; Sin-Small intestine; Te-Testis; Ov-Ovary. (B) The lysates of the above chicken tissues were separated by 10% SDS-PAGE and then stained by Coomassie brilliant blue R-250 for normalization of sample loadings. Western blotting analysis using anti-CYP2D49 antiserum was exploited to detect the expression of CYP2D49 at the protein level. (C) LMH cells were treated with rifampicin, clotrimazole, β-naphthoflavone, dexamethasone and bufuralol for 24, 48 and 72 h, respectively. Real-time PCR was used to detect the levels of *CYP2D49* mRNA. The ratio of *CYP2D49* to *β-actin* in control cells was set to 1 and the values in all treated cells were normalized relative to this value. The experiments were conducted in triplicate and the data are expressed as the mean ± SD.

To determine the distribution of CYP2D49 in chicken tissues, real-time PCR and western blotting analyses were performed on nine tissues. As shown in Fig. 3A, *CYP2D49* was ubiquitously distributed at the mRNA level in all normal tissues assayed. It was predominantly expressed in the liver, kidney and small intestine, with lower transcription levels in the brain, lung, heart, spleen, testis and ovary. Using the generated anti-CYP2D49 antiserum, the expression of CYP2D49 at the protein level was further analyzed. As shown in Fig. 3B, CYP2D49 protein exhibited similar expression patterns to its transcription patterns, with protein expression occurring predominantly in the liver, kidney and small intestine, along with weak expression in the testis and ovary. If extending the exposure time, weaker bands could be detected in the brain, lung, heart and spleen (data not shown).

To investigate the inductive properties of chicken CYP2D49 *in vitro*, Leghorn male hepatocellular carcinoma epithelial (LMH) cells were treated with four representative CYP inducers (rifampicin, clotrimazole, β-naphthoflavone and dexamethasone) and a candidate substrate of CYP2D49 (bufuralol). As shown in Fig. 3C, no induction was detectable after the treatments with the five drugs at three time points; this finding demonstrates that CYP2D49 in the liver is not inducible.

### Enzymatic properties of recombinant CYP2D49 protein

**Figure 4:**
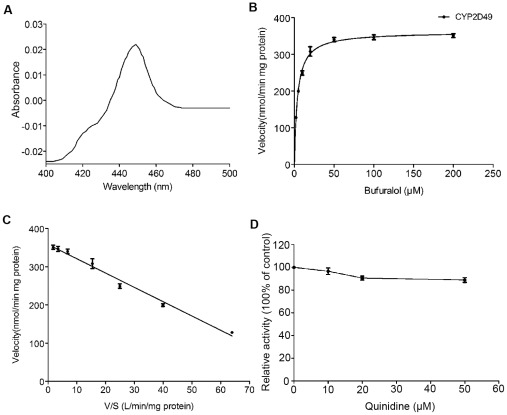
Enzymatic properties of recombinant CYP2D49 protein. (A) Reduced CO-difference spectrum of recombinant CYP2D49 (solubilized membrane fraction). The spectrum was measured in 200 mM Tris-HCl buffer (pH 7.4) containing 40% glycerol and 2 mM EDTA. No absorption at approximately 450 nm was observed in the solubilized membrane fraction of *E. coli* cells transformed with the corresponding empty vector. (B) Michaelis–Menten plot and (C) Eadie–Hofstee plot of the bufuralol 1′-hydroxylation activity of recombinant CYP2D49 enzyme. The incubation and reaction were processed essentially as described under “Materials and methods” and the enzyme kinetic parameters of CYP2D49 were determined by HPLC. Each point and bar represents the mean ± SD of three replicates. (D) Inhibitory effect of quinidine on the bufuralol 1′-hydroxylation activity of recombinant CYP2D49 enzyme. For quinidine inhibition studies, the incubation were performed by pre-incubating purified CYP2D49 protein for 5 min at 37°C with 100 µM bufuralol and varying concentrations of quinidine. The reactions were then started by the addition of NADPH. The data detection and analysis were processed essentially as described under “Materials and methods”. Each point and bar represents the mean ± SD of three replicates.

The solubilized membrane fraction of *E. coli* cells transformed with pCWOri-*CYP2D49* showed typical reduced CO-difference spectra that exhibited Soret peaks at 450 nm (Fig. 4A). However, no absorption at 450 nm was observed in the solubilized membrane fraction of *E. coli* cells transformed with the corresponding empty vector (date not shown). These results indicate that partial CYP2D49 proteins heterologously expressed in *E. coli* form holoenzymes and exhibit natural catalytic activities. To further determine the enzymatic properties of CYP2D49, metabolic assays of the purified CYP2D49 protein were performed using the typical human CYP2D6 substrates, bufuralol and debrisoquine [29]. As shown in Fig. 4B and 4C, recombinant CYP2D49 protein exhibited bufuralol 1′-hydroxylation activity. The formation of 1′-hydroxybufuralol was observed to follow simple Michaelis–Menten kinetics (Fig. 4B). The *Km* value was calculated as 3.948±0.232 µM [mean ± standard deviations (SD)] and the *Vmax* value was calculated as 361.3±4.261 nmol min^−1^ mg^−1^ protein (mean ± SD) (r^2^ = 0.99; Fig. 4B). In addition, Eadie–Hofstee plot analysis revealed that the activity of recombinant CYP2D49 enzyme followed a monophasic kinetic pattern (r^2^ = 0.97; Fig. 4C). These results demonstrate that the purified CYP2D49 is enzymatically active and can efficiently metabolize bufuralol. However, we did not detect any catalytic activity of the recombinant CYP2D49 protein toward debrisoquine (data not shown).

Quinidine is a potent inhibitor of human CYP2D6 and has been widely used as a probe of CYP2D inhibition in other animal species [13], [22], [30]. However, low concentrations of quinidine showed a negligible or low inhibitory effect toward the bufuralol 1′-hydroxylation activity of CYP2D49 (data not shown). Though increasing the concentration of quinidine from 10 µM to 50 µM, the inhibitory effect is still weak (Fig. 4D).

### Enzymatic activities of six CYP2D49 variants towards bufuralol

**Figure 5:**
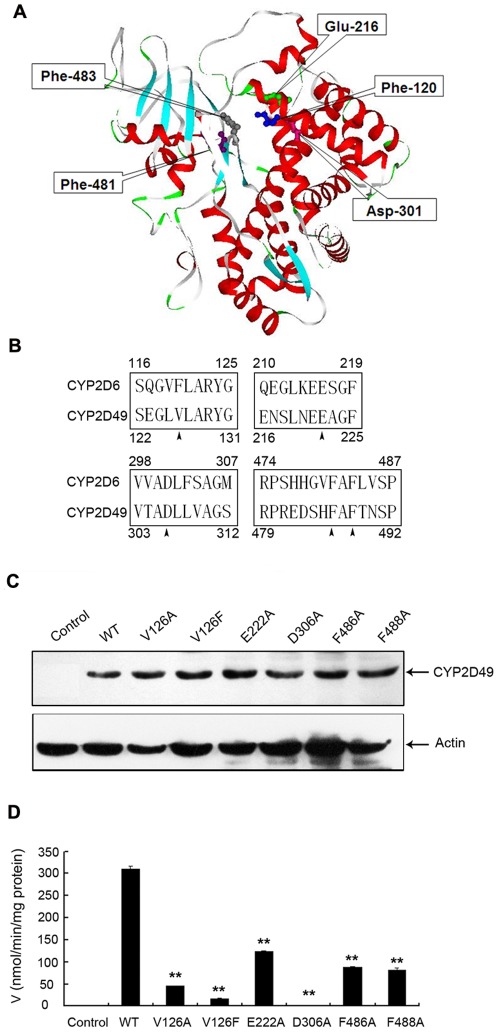
Enzymatic activities of six CYP2D49 variants towards bufuralol. (A) Crystal structure of human CYP2D6. The conformation was constructed based on the crystallographic data of CYP2D6 (2F9Q) obtained from Protein Data Bank and drawn using Accelrys ViewerLite Version 5.0. Amino acid residues at positions 120, 216, 301, 481 and 483 are in ball-and-stick form. (B) Alignment of the partial amino acid sequences of human CYP2D6 and chicken CYP2D49. The number at the top is for human CYP2D6; the number at the bottom is for chicken CYP2D49. The arrowheads show the amino acid residues to be substituted. (C) Functional expression and detection of WT CYP2D49 and its variants. S9 fractions from Hela cells which were transfected with empty vector, pcDNA-*CYP2D49*, pcDNA-*CYP2D49*-V126A, V126F, E222A, D306A, F486A and F488A were extracted and separated on 10% SDS-PAGE gels. Western blotting analysis was then used to confirm that transient transfection had succeeded. Blots were probed with the anti-CYP2D49 antiserum and a β-actin antibody, respectively. (D) Bufuralol 1′-hydroxylation catalytic activities of six CYP2D49 variants. S9 fractions of Hela cells which were transfected with empty vector, WT CYP2D49 and six CYP2D49 mutants were incubated with bufuralol as described in “Materials and methods”. Metabolites produced in the reactions were analyzed by HPLC. The data are represented as nanomoles of metabolite/min/microgram of protein. The data shown are derived from a representative experiment reported as the mean (n = 3) ± SD. Differences between the WT and mutant protein samples are significant when ** *p*<0.01.

Studying the influence of key amino acid residues on the catalytic activity of an enzyme may shed light on its catalytic mechanism. In human CYP2D6, amino acid residues such as Phe-120, Glu-216, Asp-301, Phe-481 and Phe-483 have been proposed to be involved in the substrate recognition and binding (Fig. 5A). To investigate whether the above five amino acid residues are also important for the bufuralol 1′-hydroxylation activity of CYP2D49, site-directed mutagenesis studies were conducted. We first aligned partial amino acid sequences of chicken CYP2D49 to that of human CYP2D6 and found that CYP2D49 owned the same amino acid residues as human CYP2D6 at all the sites except for the position 126, which is replaced by a valine residue (Fig. 5B). We then prepared plasmids encoding wild-type and mutant CYP2D49 (pcDNA-*CYP2D49*-V126A, V126F, E222A D306A, F486A and F488A) and transiently transfected them into Hela cells. Western blotting analysis was used to determine whether the transient transfection was successful. As shown in Fig. 5C, the transient transfection with WT and mutant CYP2D49 plasmids led to the significant over-expression of proteins with an estimated molecular mass of 55 kDa; these proteins were not detectable in control cells. The amount of CYP2D49 and its mutants detected was normalized against the detected amount of β-actin (data not shown). Furthermore, the bufuralol 1′-hydroxylation activities of the over-expressed WT and variant CYP2D49 proteins were determined by incubating bufuralol with S9 fractions from Hela cells which were transfected with empty vector, WT CYP2D49 and six CYP2D49 mutants. As shown in Fig. 5D, the WT CYP2D49 protein exhibited distinct bufuralol 1′-hydroxylation activity comparing to the control. However, significant decreases in the bufuralol 1′-hydroxylation activity were observed for all the variants, especially for the D306A variant (Fig. 5D). The bufuralol 1′-hydroxylation activities [V(nmol/min/mg protein)] of the WT CYP2D49 and the V126A, V126F, E222A, D306A, F486A and F488A variants were 309.77±6.14, 44.91±1.09, 18.17±0.53, 123.71±0.98, 2.07±0.70, 88.51±1.24 and 82.38±4.70 (mean ± SD), respectively, which indicates that these amino acid residues may play important roles in the bufuralol 1′-hydroxylation activity of chicken CYP2D49.

### Circular dichroism spectroscopy analysis

**Figure 6:**
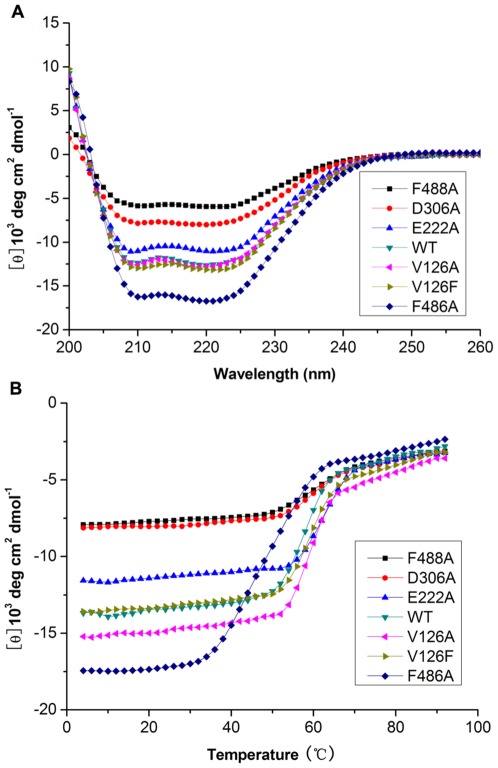
Physicochemical analyses of WT CYP2D49 protein and its variants. (A) The CD spectroscopy was used to analyze the secondary structures of the WT CYP2D49 protein and its variants. Proteins purified from *E. coli* were assayed at a final concentration of 5 µM in 0.1 M potassium phosphate (pH 7.4). The CD spectra were acquired at 4°C on the Chirascan. (B) The thermal melt was monitored by CD spectroscopy using the same proteins described in Fig. 6A. Thermal denaturation was monitored at 222 nm by applying a thermal gradient of 2°C/min over the range from 4–92°C.

The circular dichroism (CD) spectroscopy analysis is an excellent tool for the rapid determination of the secondary structure and folding properties of proteins [32]. To elucidate the relationship between the function and the structure of CYP2D49 protein, we further expressed and purified the six CYP2D49 variants from *E. coli* cells and investigated the changes of their physicochemical characterizations by CD spectroscopy analyses. As shown in Fig. 6A, the CD spectra of CYP2D49 WT and its variants exhibited the characteristic signature of an α helix with minima at 222 and 208 nm. The helical contents of the V126A, V126F, E222A, D306A, F486A, F488A variants as well as the WT protein were 38.2%, 39.8%, 33.3%, 23.9%, 50.3%, 18.1% and 37.9%, respectively. The helical content of the F486A variant was much higher than that of the WT CYP2D49, whereas the helical contents of the D306A and F488A variants were much lower (Fig. 6A). In addition, based on the mean residue ellipticity at 222 nm of a 5 µM protein preparation in 100 mM potassium phosphate (pH 7.4) at 4°C, the melting temperature (Tm) of the WT CYP2D49 was 59°C (Fig. 6B). At the same concentration, all variants displayed a cooperative thermal unfolding transition with Tm between 60–62°C, except for the F486A variant, whose melting temperature was decreased to 50°C (Fig. 6B).

## Discussion

Chicken liver microsomes exhibit bufuralol 1′-hydroxylation activity, which indicates the existence of CYP2D isoenzymes in chickens [27]. However, chicken CYP2D isoforms have not been previously identified. This prompted us to search for cDNA sequences of chicken *CYP2Ds* in the NCBI database and led to the successful identification of CYP2D49, which is the first reported avian CYP2D isoenzyme. Here, we studied the genomic organization, *in vivo* and *in vitro* expression patterns and metabolic properties of CYP2D49 and we further elucidated its probable metabolic mechanism toward bufuralol.

Human *CYP2D6* is a part of the *CYP2D* gene cluster with two inactive pseudogenes, *CYP2D7P* and *CYP2D8P* [33], [34]. However, these two pseudogenes are not found in the corresponding genomic region neighboring chicken *CYP2D49*. The different numbers of genes in the *CYP2D* cluster in humans compared with the chicken are probably the results of the gene duplications of the P450s, including *CYP2D*s, that have occurred in each species during evolution [35]. To find out whether there is any other *CYP2D* existed in chickens, we used the sequence of *CYP2D49* to search for its homologues in the chicken EST and genomic database but none was found. Thus, *CYP2D49* appears to be the best candidate “orthologue” of *CYP2D6* in chickens. However, we cannot exclude the possibility that there are other CYP2Ds in chickens due to incomplete EST and genomic information.

The expression data indicate that CYP2D49, similarly to CYP2D6, is highly expressed in the liver, kidney and small intestine and modestly expressed in various other tissues [30]. The specific tissue distribution of chicken CYP2D49 suggests that CYP2D49 protein may have specific catalytic properties and play specific roles in the liver, kidney and small intestine. Based on these, we further studied the inductive characterization of CYP2D49 in the liver. In humans, CYP1A1, CYP1A2, CYP2B6, CYP2C8, CYP2C9, CYP2C19 and CYP3A4 are known to be inducible, whereas CYP2D6 is not [30]. Rifampicin, β-naphthoflavone, dexamethasone and clotrimazole are representative CYP inducers and can induce the expression of chicken CYP3A37, CYP2H1 and CYP2C45 in LMH cells, the first continuously dividing cell line of the chicken liver [26], [36], [37]. However, none of these inducers can increase the expression of CYP2D49 in LMH cells. CYPs are often induced by their own substrates to allow a dynamic adaptation to xenobiotic exposure [38]. However, the candidate substrate of CYP2D49 (bufuralol) also failed to induce the expression of CYP2D49. All these indicate that CYP2D49 is not inducible in the liver, similar to human CYP2D6. Further studies are still required to demonstrate the regulational mechanisms of the *in vivo* and *in vitro* expression patterns of CYP2D49.

Recombinant CYP2D49 protein expressed from either prokaryotic or eukaryotic systems effectively hydroxylated bufuralol, as typically observed for human CYP2D6 [29]. Moreover, the *Km* value of CYP2D49 for bufuralol 1′-hydroxylation reaction (3.948±0.232 µM) is similar to that of human CYP2D6 (4.4 µM) [39]. All these further confirm our notion that chicken CYP2D49 is an orthologue of human CYP2D6 and indicate that CYP2D49 may have similar bufuralol binding affinity to human CYP2D6. However, the *Km* value of CYP2D49 for bufuralol 1′-hydroxylation reaction is much lower than that of the chicken liver microsomes reported by Khalil *et al.*, who also investigated the kinetic parameters of dog liver microsomes for bufuralol parallel [27]. We found that the *Km* value of dog liver microsomes for bufuralol 1′-hydroxylation reaction [27] is higher than the value reported by Roussel *et al* [31]. Thus, further studies are still needed to confirm the enzymatic kinetics of chicken liver microsomes for bufuralol and the critical role of CYP2D49 in this reaction.

Clear differences exist between humans and other animal species with regard to the phase I and phase II drug metabolic reactions [30]. Though our data from the genomic organization, *in vivo* and *in vitro* expression patterns and metabolic properties of CYP2D49 indicate that chicken CYP2D49 is an orthologue of human CYP2D6, these differences are also observed between chicken CYP2D49 and human CYP2D6. For example, recombinant CYP2D49 showed inefficient debrisoquine 4′-hydroxylation activity, which is another typical representative reaction of human CYP2D6 [21]. Indeed, rat CYP2D3 and CYP2D4 are also deficient in their ability to metabolize debrisoquine, which is mainly catalyzed by CYP2D2 [10], [40]. Determining whether the chicken has lost the ability to transform debrisoquine or if other CYPs are responsible for this reaction requires further study. In addition, quinidine, a prototypical inhibitor of human CYP2D6 [13], [30], showed a negligible inhibitory effect toward the bufuralol 1′-hydroxylation activity of CYP2D49. In fact, the inhibition profiles of the rat, monkey and mouse proteins also differ from that of the human protein. Bogaards *et al.* reported a negligible (in the rat and mouse) or low (in the monkey) inhibitory effect by quinidine toward bufuralol 1′-hydroxylase catalytic activity [41].

Most substrates of CYP2D6 include an aromatic moiety and a basic nitrogen atom in their structures. Site-directed mutagenesis, computational modeling and the crystal structure of human CYP2D6 identified the importance of Phe-120, Glu-216, Asp-301, Phe-481 and Phe-483 in substrate recognition and binding [8], [9]. Glu-216 and Asp-301, which are two negatively charged residues in the active site of human CYP2D6, facilitated the binding and orientation of the ligands in the active site through the formation of an electrostatic interaction between their carboxylate group and the basic nitrogen atom of the CYP2D6 substrates [42], [43]. Additionally, the aromatic side chains of Phe-120, Phe-481 and Phe-483 interacted with the aromatic moiety of the substrate through a hydrophobic (π-π) interaction [9], [44]–[46]. Each mutation of the position 222, 306, 486 and 488 in CYP2D49 to a neutral alanine residue greatly decreased the catalytic efficiency of CYP2D49 toward bufuralol, especially for the mutation D306A. This finding indicates that these four amino acid residues are important for the bufuralol 1′-hydroxylation activity catalyzed by CYP2D49. Further studies are still needed to confirm whether the four sites in CYP2D49 function in the same way to that of human CYP2D6.

It is interesting to note that Phe-120 is not conserved in CYP2D49; rather, it is replaced by a valine residue. In fact, rat CYP2D2, CYP2D3 and CYP2D4 possess a valine instead of a phenylalanine at position 120 and exhibit normal bufuralol 1′-hydroxylation activity [47]. We then mutated this position to an alanine or a phenylalanine; both mutations decreased the catalytic activity of CYP2D49 toward bufuralol. These results demonstrate that Val-126 is also important for the catalytic activity of CYP2D49 toward bufuralol. Although Phe-120 was considered to be important in orienting the aromatic ring of most substrates with respect to the heme moiety of the enzyme, no change was found in the apparent *Km* or *Vmax* values for bufuralol oxidation catalyzed by human CYP2D6 F120A variant [45]. Thus, the phenylalanine at position 120 may be not critical to the catalytic activity of human CYP2D6 when the substrate is bufuralol. Additionally, Narimatsu *et al.* found that rat CYP2D4 V123F variant showed increased *Km* values and decreased *Vmax* values for bufuralol oxidation, indicating that Val not Phe at position 123 is important for the catalytic activity of CYP2D4 toward bufuralol [48]. They speculated that bufuralol with a basic nitrogen atom can be captured ionically by the carboxylate group of Glu-219 or Asp-304 (corresponding to Glu-216 and Asp-301 of human CYP2D6), resulting in an orientation whereby the oxidation site (1′-position) of bufuralol comes close to the heme iron. However, the mutated Phe at position 123 captures bufuralol via hydrophobic (π-π) interaction between the aromatic rings of Phe-123 and bufuralol, lowering the efficacy of the bufuralol oxidation by interfering with the interaction of other bufuralol molecules with the carboxylate group of Glu-219 or Asp-304. The CYP2D49 V126F variant may function in the same way to that of rat CYP2D4. However, further studies are still needed to explore the exact mechanism.

Furthermore, CD spectroscopy was used to analyze the effect of site-directed mutagenesis on the secondary structure of CYP2D49 and to reveal the relationship between the function and the structure of CYP2D49. All six mutations decreased the ability of CYP2D49 to metabolize bufuralol. However, only D306A, F486A and F488A variants changed the α-helical content of CYP2D49 protein and the F486A variant decreased the Tm value of CYP2D49 protein. These results demonstrate that Asp-306, Phe-486 and Phe-488 may play critical roles in maintaining the conformation of CYP2D49 protein; mutations at these sites may break the natural structure of CYP2D49 protein and lead to great decrease in the catalytic efficiency of CYP2D49 toward bufuralol. Moreover, both V126A and V126F mutations have no influence on the secondary structure and thermal stability of CYP2D49, suggesting that Val-126 may not function through maintaining the conformation of CYP2D49 protein. However, how exactly Val-126 affects the catalytic activity of CYP2D49 toward bufuralol still needs further study.

The relationship between enzymatic activity and protein structure and stability is very complicated. Any change of the structure of an enzyme could affect its catalytic activity to some extent, however, protein structure and stability is not the only factor influencing its activity. The key sites of an enzyme can also function through direct interactions with the substrates. Besides breaking the natural structure of CYP2D49 protein, the D306A variant may destroy the electrostatic interaction between carboxylate group of Asp-306 and the basic nitrogen atom of bufuralol, which inhibited the oxidation site (1′-position) of bufuralol coming close to the heme iron and resulted in great decrease of the catalytic efficiency of CYP2D49 toward bufuralol. However, the F486A variant may mainly change the conformation of CYP2D49 and lead to the decrease in the catalytic efficiency of CYP2D49 toward bufuralol to a less extent. All these speculations still need more experimental evidences.

In conclusion, we have found a novel and also the first *CYP2D* gene in the chicken. Studies of its sequence, tissue expression pattern, inductive properties and metabolic characteristics show that CYP2D49 can catalyze the 1′-hydroxylation of bufuralol and may be a counterpart to human CYP2D6 in chickens. Further investigations of the metabolic mechanism of CYP2D49 identified the importance of Val-126, Glu-222, Asp-306, Phe-486 and Phe-488 in keeping the enzymatic activity of CYP2D49 toward bufuralol as well as the importance of Asp-306, Phe-486 and Phe-488 in maintaining the conformation of CYP2D49 protein. The current study is only the first step in characterizing the metabolic mechanism of CYP2D49; further studies are still required.

## Materials and Methods

### Ethics statement

This study was carried out in strict accordance with the recommendations in the Regulations for the Administration of Affairs Concerning Experimental Animals of Guangdong Province, China. The protocol was approved by the Committee on the Animal Care and Use of Laboratory Animal Center of Sun Yat-sen University (Production Permit Number: SCXK 2004-0011; Use Permit Number: SYXK 2007-0081). All efforts were made to minimize suffering.

### Chemicals and reagents

Bufuralol hydrochloride, 1′-hydroxybufuralol, debrisoquine and 4′-hydroxydebrisoquine were purchased from TRC (North York, Ontario, Canada). NADPH-P450 reductase, cytochrome b5, NADPH, quinidine, rifampicin, β-naphthoflavone, clotrimazole and dexamethasone were obtained from Sigma-Aldrich (St. Louis, MO, USA). Acetonitrile (ACN) and water used for high-performance liquid chromatography (HPLC) were obtained from Thermo Fisher Scientific (Fairlawn, NJ, USA) and Milli-Q Ultra-purification Systems (Millipore, Bedford, MA, USA), respectively. All the other chemicals and reagents used were of analytical grade.

### Animals and cell culture

Three-yellow broilers (three males and three females, 7–8 weeks old, 1.2–1.5 kg) were purchased from the College of Veterinary Medicine at South China Agricultural University (SCAU). Chicken tissues for RNA and protein preparation were collected, snap-frozen in liquid nitrogen and stored at −80°C until use. BALB/c mice (five males, 5–6 weeks old) were used to generate polyclonal anti-CYP2D49 antiserum and purchased from the Laboratory Animal Center of Sun Yat-sen University (SYSU). The chickens and mice were fed commercial standard diets and were allowed access to water *ad libitum* to ensure the absence of therapeutic or illicit treatments before slaughtering.

Hela cells (ATCC, CCL-2) and LMH cells (ATCC, CRL-2117) were maintained at 37°C in Dulbecco's Modified Eagle's Medium (DMEM) and William's E medium (Sigma-Aldrich, St. Louis, MO, USA), respectively, that were supplemented with 10% fetal bovine serum (FBS), 100 U/ml penicillin and 100 µg/ml streptomycin. For induction, the LMH cells were treated with concentrations of 25, 10, 10, 25 and 100 µM, respectively, of rifampicin, clotrimazole, β-naphthoflavone, dexamethasone and bufuralol, which were dissolved in DMSO (Sigma-Aldrich, St. Louis, MO, USA). After treatment for 24, 48 and 72 h, the cells were collected for RNA extraction. The control cells were incubated in equal solvent concentrations.

### cDNA cloning and the construction of plasmids

Total RNA from the chicken liver was extracted with the SV Total RNA Isolation System (Promega, Madison, WI, USA) and first-strand cDNA was synthesized using SMART MMLV Reverse Transcriptase (Promega, Madison, WI, USA) according to the manufacturer's instructions. The cDNA encoding chicken *CYP2D49* was amplified by polymerase chain reaction (PCR) from the single-stranded cDNA template using the following primers: 5′-CGGGGAGGGGAGCAGGAGAA-3′ (sense) and 5′-CGCAGGAACTCAGGACTAAAAC-3′ (antisense). These primers were designed based on the nucleotide sequence of the flanking region of the chicken finished cDNA clone CHEST77m14 (GenBank accession no. CR354312.1). The PCR product was cloned into the pMD20-T vector (TaKaRa, Qingdao, China) and verified by DNA sequencing. The deduced sequence was submitted to the P450 nomenclature committee for name designation and then to GenBank (accession no. AEZ51809).

To allow functional expression in *E. coli*, the N-terminal coding region of CYP cDNA requires a modification, for which we selected *ompA*+2 according to the Cytochrome P450 Protocols [49]. Briefly, a cDNA fragment encoding the bacterial *ompA* leader sequence (21 amino acid residues) and two additional spacer amino acid residues (Leu-Glu) were fused to the *CYP2D49* encoding sequence by PCR. The *ompA*-*CYP2D49* cassette was then inserted into the *Not* I*/Kpn* I sites of the pCWOri+ vector (pCWOri-*CYP2D49*). To allow eukaryotic expression, the open reading frame (ORF) region of *CYP2D49* was inserted into the *Not* I/*Hind* III sites of the pcDNA™3.1/myc-His (−) A (Invitrogen, Carlsbad, CA, USA) vector (pcDNA-*CYP2D49*). Six mutants (pcDNA-*CYP2D49*-V126A, V126F, E222A, D306A, F486A and F488A) were prepared from pcDNA-*CYP2D49* using the QuikChange Site-directed Mutagenesis Kit (Invitrogen, Carlsbad, CA, USA). The mutant cDNAs from pcDNA™3.1/myc-His (−) A were then subcloned into the pCWOri+ vector using primers containing *Not* I*/Kpn* I sites. All plasmids were verified by DNA sequencing.

### Recombinant protein expression and preparation of anti-CYP2D49 antiserum

The expression plasmid pCWOri-*CYP2D49* was transformed into DH5α competent cells. A single clone was randomly picked and cultured at 37°C for 5–7 h in LB media containing 100 mg/L ampicillin first and then used to inoculate modified TB media (12 g/L bactotryptone, 24 g/L yeast extract, 2 g/L bactopeptone and 4 ml/L glycerol) containing 100 mg/L ampicillin and 1.0 mM thiamine with supplementation of trace elements. IPTG was added at a final concentration of 1 mM when the OD_600_ of the cell culture reached 0.7–0.8 and the expression of recombinant CYP2D49 was then induced at 30°C for 36 h. The cultures were then chilled on ice and centrifuged at 2,800×*g* for 20 min at 4°C. The cell pellet was re-suspended in 100 mM Tris acetate buffer (pH 7.6) containing 500 mM sucrose and 0.5 mM EDTA and the suspension was then diluted with an equal volume of ice-cold water. Lysozyme was added to the re-suspended cells at a final concentration of 0.25 mg/ml. The suspension was incubated at 4°C for 45 min with agitation. The spheroplasts were pelleted at 2,800×*g* for 20 min at 4°C and re-suspended in 100 mM potassium phosphate buffer (pH 7.6) containing 6 mM magnesium acetate, 20% glycerol (v/v) and 0.1 mM DL-dithiothreitol. The suspensions were sonicated on ice and centrifuged at 12,000×*g* for 20 min at 4°C. The supernatants were pipetted into ultracentrifuge tubes and centrifuged at 220,000×*g* for 60 min at 4°C. The membrane pellets were dissolved in ice-cold MCAC-0 buffer (pH 7.4) containing 20 mM Tris, 500 mM NaCl, 1% Triton X-100 (v/v) and 20% glycerol (v/v). The solubilized membrane fraction containing CYP2D49 was loaded onto a nickel-charged NTA-agarose column (HisTrap HP, 5 ml, GE, USA) and purified by fast protein liquid chromatography (FPLC) (AKTA purifier, GE, USA) according to the manufacturer's instructions. The same methods were used to express and purify the six CYP2D49 variants (pCWOri-*CYP2D49*-V126A, V126F, E222A, D306A, F486A and F488A).

In metabolic assays, the reduced CO-difference spectrum of recombinant CYP2D49 (solubilized membrane fraction) was measured spectrophotometrically according to the Cytochrome P450 protocols [49]. The protein concentrations were determined by the Bradford method. For CD spectra, all purified proteins were further dialyzed in 100 mM potassium phosphate buffer (pH 7.4) overnight.

Purified CYP2D49 protein was also used to immunize mice to raise polyclonal anti-CYP2D49 antiserum according to the protocol of a previous report [50]. Briefly, prior to a course of immunization, approximately 0.2 ml of blood was collected from the mouse to provide a source of pre-immune antiserum. One week later, approximately 300 µg of purified CYP2D49 protein emulsified in complete Freund's adjuvant was injected subcutaneously into the scruff of the mice. After the first injection, booster injections were performed three times at two-week intervals with the same antigen amount but using incomplete Freund's adjuvant (Sigma-Aldrich, St. Louis, MO, USA). The mice were then exsanguinated. The blood was stored at 25°C overnight and then centrifuged at 3,000×*g* for 10 min at 4°C. The antisera were collected and stored at −80°C until use.

### RNA isolation and real-time PCR

The total RNA from LMH cells and chicken tissues was extracted using TRIZOL Reagent (Invitrogen, Carlsbad, CA, USA) and the SV Total RNA Isolation System (Promega, Madison, WI, USA), respectively. First-strand cDNA was synthesized using random primers and M-MLV reverse transcriptase (Promega, Madison, WI, USA). The primers used in real-time PCR were as follows:

*CYP2D49*-F: 5′-GGCAAAGGGTAAGGAGGCT-3′;

*CYP2D49*-R: 5′-TGACGGCATTGGTGTAGGG-3′;

*18s rRNA*-F: 5′-GAGAAACGGCTACCACATCC-3′;

*18s rRNA*-R: 5′-CACCAGACTTGCCCTCCAA-3′;

*β-actin*-F: 5′-GGCTGTGCTGTCCCTGTA-3′;

*β-actin*-R: 5′-CGGCTGTGGTGGTGAAG-3′.

Real-time PCR was performed on an Opticon 2 real-time PCR system (Bio-Rad, Hercules, CA), according to the manufacturer's recommendations. Reactions were performed in a 20-µl volume containing SYBR Green I Dye. The cycling parameters were 94°C for 2 min, followed by 33 cycles of 94°C for 20 s, 55°C for 20 s and 72°C for 20 s. All samples were analyzed in triplicate and the expression of the target gene was calculated relative to the expression of *18s rRNA* (or *β-actin*) according to the 2^−ΔCT^ (or 2^−ΔΔCT^) method [51].

### Western blotting analysis

Protein samples from chicken tissues, S9 fractions of transiently transfected Hela cells and induced bacteria were separated on 10% SDS-PAGE gels and then electrophoretically transferred to PVDF membranes (PALL, Ann Arbor, MI, USA). The membranes were blocked with freshly prepared TBST buffer (25 mM Tris-HCl pH 7.5, 150 mM NaCl and 0.1% Tween-20) containing 5% nonfat dry milk for 1 h at room temperature. The membranes were then incubated for 1 h with the primary antibody in TBST buffer containing 1% milk, washed three times for 10 min with TBST, incubated with the secondary antibody for 1 h at room temperature and then washed for another 30 min with TBST buffer. The bands were developed for detection with the LumiGLO® Chemiluminescent Substrate Kit (CST, Beverly, MA, USA) according to the manufacturer's instructions. To confirm the specificity of the anti-CYP2D49 antiserum, the membranes were incubated with five anti-CYP2D49 antiserum from five mice, respectively, that had been pre-adsorbed with excess antigen (purified CYP2D49-Myc-His-Tag fusion protein) at 37°C for 1 h in TBST buffer containing 1% milk. The specific one was used in the left experiments. When studying the enzymatic properties of CYP2D49 variants in Hela cells, β-actin was co-analyzed as the standard on each gel for the quantification of CYP2D49 and its variants. Chemiluminescence was quantified using the quantity tool of Image Lab™ software (Bio-Rad, Hercules, CA, USA). The dilutions employed for the antibodies were as follows: polyclonal anti-CYP2D49 antiserum at 1∶1500; β-actin (C4) (sc-47778, Santa Cruz Biotechnology, Santa Cruz, CA, USA) at 1∶1000; c-Myc (9E10) (sc-40, Santa Cruz Biotechnology, Santa Cruz, CA, USA) at 1∶1000; HRP-rabbit anti-mouse IgG (Gamma) (Invitrogen, Carlsbad, CA, USA) at 1∶4000.

### Enzymatic kinetics and inhibition assays

Bufuralol is a typical substrate of the CYP2D isoforms. The level of 1′-hydroxybufuralol, a major metabolite of bufuralol, is often measured as an index of CYP2D activity and/or levels [13]. The bufuralol 1′-hydroxylation activity of CYP2D49 was determined by incubating bufuralol with the purified CYP2D49 protein. The incubation mixture contained 100 µg of CYP2D49 protein (protein inactivated by heating for 10 min was used as the control), 0.2 µM NADPH-P450 reductase, 0.1 µM cytochrome b5, 0.02 mg/ml liposome (1, 2-dioleoyl-sn-glycero-3-phosphocholine, 1, 2-didodecanoyl-rac-glycero-3-phosphocholine and 1, 2-diacyl-sn-glycero-3-phospho-L-serine mixture), 10 mM MgCl_2_, 2–200 µM bufuralol and 100 mM potassium phosphate buffer (pH 7.4) in a final volume of 250 µl. After a 3-min pre-incubation at 37°C in a shaking water bath, the reaction was started by adding NADPH and further incubated at 37°C for 10 min. After the reaction was stopped by the addition of 20 µl of 60% HClO_4_ aqueous solution and vortexed, the reaction mixture was centrifuged at 12,000×*g* for 5 min at room temperature. An aliquot (20 µl) of the supernatant was subjected to HPLC analysis using a Waters 2695 HPLC System (Waters Alliance, Milford, MA) equipped with a Waters 2475 fluorescence detector. To separate bufuralol and the bufuralol 1′-hydroxylation product, the samples were injected onto a Hypersil BDS C18 column (4.6×250 mm I.D; particle size 5 µm; Elite, Dalian, China) equipped with an Analytical Guard Column (3.0×20 mm I.D.; particle size 5 µm; Phenomenex). The mobile phase consisted of 20 mM perchloric acid (pH 2.5) and acetonitrile (65∶35, v/v). The chromatograph was operated at a flow rate of 1 ml/min at room temperature, with fluorescence detection at 252/302 nm (excitation/emission). A quantitative analysis of the reaction products was performed using the peak area.

Debrisoquine is a classical substrate for the CYP2D isoforms and, similar to bufuralol, serves as a probe of CYP2D activity. The amount of 4′-hydroxydebrisoquine transform from debrisoquine is used to judge the enzymatic activity of CYP2D isoforms [52]. The incubation conditions were similar to those used with bufuralol and the detection of 4′-hydroxydebrisoquine was performed according to a published method [21].

The experiments involving inhibition by quinidine were performed by pre-incubating purified CYP2D49 protein for 5 min at 37°C with 100 µM bufuralol and varying concentrations of quinidine. The reactions were then started by the addition of NADPH. The reactions were incubated and the reaction products were detected as described above.

### Expression and enzymatic properties of CYP2D49 variants in Hela cells

The Hela cell line was selected as the recipient cell line because of its high transient transfection efficiency and its low endogenous levels of drug metabolism. Before transfection, the Hela cells were grown overnight to 80% confluence in a 10-cm dish. For each dish, 24 µg of the expression constructs (pcDNA-*CYP2D49*, one of the six mutant constructs or the empty vector) was combined with 60 µl of Lipofectamine 2000 (Invitrogen, Carlsbad, CA, USA) in 3 ml of FBS-free DMEM. The transfections were performed in 6 ml of FBS-free DMEM for 6 h. The culture medium was then replaced with 12 ml of fresh medium (10% FBS in DMEM). Forty-eight hours after transfection, the cells grown in the culture medium were rinsed with phosphate-buffered saline (PBS, pH 7.4), scraped and collected in 100 mM potassium phosphate buffer (pH 7.4). They were then sonicated with five pulses at 40 W for 5 s at 10 s intervals. The S9 fractions containing the cytosol and microsomes are frequently used in assays to measure the metabolism of drugs and other xenobiotics. Therefore, the cell homogenate was centrifuged at 9,000×*g* at 4°C for 20 min to isolate the S9 fraction, which was then carefully transferred to a clean tube for western blotting analyses or assays of enzymatic activity. The protein concentration of S9 fractions were estimated by the Bradford method.

The bufuralol 1′-hydroxylation activity of each S9 fraction was determined according to a published method [21] with a slight modification. Briefly, in a brown glass conical tube (2 ml), a 250-µl incubation mixture was prepared that contained 1.0 mg of protein, 10 mM MgCl_2_, 1 mM NADPH and 100 µM bufuralol in 100 mM potassium phosphate buffer (pH 7.4). After a 3-min pre-incubation at 37°C,the reaction was started by adding NADPH and further incubated at 37°C for 10 min. The detection of 1′-hydroxybufuralol was performed as described above.

### Circular dichroism spectroscopy

All CYP2D49 variants were assayed at a concentration of 5 µM in 100 mM potassium phosphate buffer (pH 7.4). The circular dichroism (CD) spectra of these samples were obtained using the Chirascan (Applied Photophysics Limited, Leatherhead, Surrey, UK) at 4°C using a 1.0-nm bandwidth, 1-mm cell, 1.0-nm step, 0.5-s time-per-point and 1.0-min time internal. The thermal denaturation was monitored at 222 nm by applying a thermal gradient of 2°C/min over the range from 4 to 92°C. The value for the buffer alone was measured and subtracted from the protein spectra. The CD spectra of all of the proteins were acquired as ‘ellipticity’ in millidegrees *θ*. The data were converted to the mean residue ellipticity ([*θ*] in degree cm^2^ dmol^−1^), as described previously [32]. The [*θ*] at 222 nm of −33000 deg cm^2^ dmol^−1^ was regarded as representing a 100% helical conformation. The helical content of the proteins was estimated by dividing the value of −33000 cm^2^ dmol^−1^ by the [*θ*] value at 222 nm [53].

### Data analysis

The standard curve of 1′-hydroxybufuralol was calculated using a linear least-squares regression analysis (Microsoft® Excel, 2003). The estimations and statistical analyses of the enzyme kinetic and inhibition parameters were performed using GraphPad Prism® software, version 5. Significant differences were evaluated by ANOVA (significance was defined as *p*<0.05).

The conformation of CYP2D6 was constructed based on the crystallographic data of CYP2D6 (2F9Q) obtained from Protein Data Bank (http://www.rcsb.org/pbd/) and drawn using Accelrys ViewerLite Version 5.0.
